# Dust evolution, a global view I. Nanoparticles, nascence, nitrogen and natural selection … joining the dots

**DOI:** 10.1098/rsos.160221

**Published:** 2016-12-14

**Authors:** A. P. Jones

**Affiliations:** Institut d’Astrophysique Spatiale, CNRS, Univ. Paris-Sud, Université Paris-Saclay, Bât. 121, 91405 Orsay cedex, France

**Keywords:** interstellar medium, interstellar dust, interstellar molecules

## Abstract

The role and importance of nanoparticles for interstellar chemistry and beyond is explored within the framework of *The Heterogeneous dust Evolution Model for Interstellar Solids* (THEMIS), focusing on their active surface chemistry, the effects of nitrogen doping and the natural selection of interesting nanoparticle sub-structures. Nanoparticle-driven chemistry, and in particular the role of intrinsic epoxide-type structures, could provide a viable route to the observed gas phase OH in tenuous interstellar clouds en route to becoming molecular clouds. The aromatic-rich moieties present in asphaltenes probably provide a viable model for the structures present within aromatic-rich interstellar carbonaceous grains. The observed doping of such nanoparticle structures with nitrogen, if also prevalent in interstellar dust, could perhaps have important and observable consequences for surface chemistry and the formation of precursor pre-biotic species.

## 1. Introduction

The smaller end of the interstellar dust size distribution, which extends from micrometres down to sub-nanometre sizes, is at the heart of many important astrophysical processes, such as: the ultraviolet extinction that shields molecules from photo-dissociation, the origin of the IR emission bands, the anomalous microwave emission assumed to come from spinning nanoparticles, the heating of the gas through photoelectric emission and the catalytic formation of molecular hydrogen. As has been long appreciated, the formation of H_2_, via H atom sticking, accommodation, surface mobility and recombination on grain surfaces [1,2], is the major driving force for most interstellar chemistry.

Given that nanoscience studies encompass particles with sizes up to several hundreds of nanometres, it would appear that the majority of interstellar grains should be considered as nanoparticles. However, here we prefer to restrict our definition of nanoparticles to particles with sizes of the order of 1 nm, which exhibit properties significantly different from larger grains and the parent bulk materials [3,4]. Even with this restrictive definition, nanoparticles have been rather widely considered within the narrower confines of dust astrophysics.

Some of the earliest studies to consider nanoparticle physics, even though it was not labelled as such at the time, were attempts to explain the origin of the interstellar ultraviolet extinction bump, centred at ≃217.5 nm [5], in terms of the hydrogenation of small carbonaceous grains [6,7]. More recently, work on the likely origin of the extinction bump [8–11] has been extended, both experimentally and theoretically, to explorations of the likely contributions of carbonaceous nanoparticles to interstellar extinction [12,13], the origin of the infrared emission features [4,13–16], the observed extended red emission (ERE) and blue luminescence (BL) [17–19], the diffuse interstellar bands (DIBs) [19,20], and polyyne sub-structures within carbon nanoparticles [21]. Carbonaceous nanoparticles are particularly interesting, and perhaps rather fickle beasts, because their composition and structure (principally, a mix of aromatic, olefinic and aliphatic hydrocarbon domains) depend on both their formation and their current environment. In particular, whether they are predominantly aromatic or aliphatic, and the nature of aromatization and aliphatization processes has been much debated in the astronomical literature [4,8,9,22–28]. Such grain structures have also been dubbed mixed aromatic–aliphatic organic nanoparticles or MAONs [29–31].

Interstellar nanoparticles also come in many other hypothesized guises. For instance, the possible contribution of silicon nanoparticles to interstellar extinction has been considered [32,33] and it was postulated that they could be the carriers of the observed ERE [34–37] and BL [38]. Iron, as observed remotely within extraterrestrial dusts [39–41] and by direct analysis in the form of nanoparticles as inclusions within larger silicate grains [42,43], has been proposed as an important contributor to interstellar dust extinction [44]. The magnetic properties of iron and iron oxide nanoparticles as free-flying grains or as inclusions within larger grains could also have important consequences for dust polarization and the anomalous microwave emission attributed to spinning dust [45–47].

Perhaps, one of the closest analogues for interstellar nanoparticles is actually to be found rather close to home and in the shape of the nanoparticles recovered from meteorites, which should serve as realistic and viable models for interstellar grains [48].

Some would perhaps also consider that the hypothesized interstellar polycyclic aromatic hydrocarbons (PAHs) [49] are also nanoparticles, as indeed they must be. However, it appears that the PAH hypothesis, as attractive as it may seem after all the efforts invested in it, is not without some difficulties [4,13]. In our work, we consider that structures analogous to PAHs are indeed to be found within interstellar dust but that, rather that existing as free-flying species, they are the aromatic domains intrinsic to hydrogenated amorphous carbon materials [4,50].

An understanding of the peculiarities of nanoparticle physics within the interstellar medium (ISM) is therefore absolutely essential but, despite all of the efforts mentioned above, this has not yet been fully recognized or received its due attention. In most dust studies, the physical properties of nanoparticles are simply treated using a top-down extrapolation of the properties of bulk materials extended down to nanometre sizes [6,7,51–54]. Such an approach is over-simplistic because the size-dependent properties of interstellar analogue materials [3] can help us to self-consistently understand and explain many of the critical observable properties of dust in the ISM [4,6,7,13,55]. An alternative approach has been to start from molecular properties and extend those properties up to macro-molecular nanoparticle sizes, which is the basis of the interstellar PAH hypothesis [49]. This approach is also not without problems because, for example:

— it is not clear that large planar, polycyclic aromatic species will retain their inherent structure in the harsh environments of the ISM [56–62];
— the DIB correlation with dust is stronger than with small radicals (e.g. C_2_, C_3_, CN, CH, …), implying that interstellar chemistry is more consistent with a top-down process than with bottom-up formation [63,64]; and
— the extrapolation of PAH absorption cross-sections to nanoparticle dimensions appears to be somewhat problematic [4,13].

Here, some of the consequences of adopting a more realistic approach to the chemistry and physics of nanoparticles are explored. The paper is organized as follows: §2 briefly describes the fundamentals of the assumed dust model (THEMIS), §3 focuses on the nature of interstellar nanoparticles, §4 considers the active chemistry of nanoparticle surfaces, §5 investigates the role of nitrogen atom doping in carbonaceous nanograins, §6 speculates on the nature and natural selection of possibly pre-biotic aromatic-type moieties and §7 concludes this work.

## 2. The Heterogeneous dust Evolution Model for Interstellar Solids

The ideas presented here and in the accompanying papers [64,65] arise from a re-consideration of the nature of interstellar dust within the framework of *The Heterogeneous dust Evolution Model for Interstellar Solids* (THEMIS) [13,44,66]. This new dust modelling approach makes the fundamental assumption that the interstellar silicate and carbonaceous dust populations, both essentially completely amorphous in nature, cannot be completely segregated in the ISM because of the cycling of matter, especially carbon, between the gas and dust phases. This re-cycling is due to destructive processing in energetic environments such as shocks and photo-dissociation regions with intense stellar UV/EUV radiation fields. The result is that amorphous silicate grains are mixed with a carbonaceous dust component,^1^ most probably in core/mantle (CM) structures [13,55,67–69,]. The foundation of the THEMIS dust modelling approach is its diffuse ISM dust model [13,44], which comprises the following grain and core/mantle (CM) grain structures and compositions:

— a steep power-law distribution of hydrogen-poor amorphous carbon, a-C, nanoparticles (radii 0.4≤a≲20 nm) with strongly size-dependent optical properties and most of the mass in the smallest nanoparticles, i.e. d*n*(*a*)/d*a*∝*a*^−5^;
— a lognormal distribution (*a*≃10–3000 nm, *a*_peak_≃160 nm) of large hydrogenated amorphous carbon, a-C(:H), CM grains with UV photolysed a-C surface layers (mantle depth ≃20 nm) surrounding hydrogen-rich amorphous carbon cores, a-C:H; and
— a lognormal distribution (*a*≃10–3000 nm, *a*_peak_≃140 nm) of large amorphous silicate grains, a-Sil, of olivine-type and pyroxene-type composition with Fe metal and FeS nanoparticle inclusions, a-Sil_Fe,FeS_, with a-C mantles (depth ≲10 nm) formed by carbon accretion and/or by the coagulation of small a-C particles onto their surfaces.

This diffuse ISM dust model is consistent with the interstellar IR-UV extinction, IR emission bands, FIR-mm thermal emission and dust scattering (albedo) and the observed variations of these properties in the diffuse ISM and also in the transition from diffuse to dense regions [13,44,70–74].

A key aspect of the THEMIS dust model is the hydrogenation state of the grains, and in particular of the a-C(:H) nanoparticles, a matter that was long ago [6,7,69,75] and more recently [55] also given careful consideration. In the earlier works, it was proposed that hydrogen plays a key role in determining nature of the 217.5 nm UV bump, which was assumed to arise in hydrogen-free, carbonaceous nanoparticles (a≲6 nm) [6,7], graphitized by UV starlight in the diffuse ISM on a time-scale of ≈10^8^ yr [7]. Conversely, larger grains should retain their hydrogen and would therefore not exhibit a 217.5 nm feature [6]. Within the framework of this early modelling, the UV bump was predicted to depend on the local conditions [6,7]; being strong and narrow in clouds with a high UV flux and a low H atom density and weakening and broadening in denser clouds due to H atom accretion and grain hydrogenation [6,7]. With these early models the small grains were predicted to be H-poor due to hydrogen being driven off by stochastic heating events [6,7]. Early work also showed that the carbon in the diffuse ISM was most likely to be in the form of carbonaceous mantles accreted onto silicate-oxide cores and that the sp^3^/sp^2^ carbon atom ratio in these mantles should be a function of the grain mantle temperature [75]. Cold mantles would be rich in sp^3^ diamond-like carbon and show a 3.4 μm absorption band towards highly reddened field stars (*A*_V_∼10–40), which is consistent with observations provided that the mantles have C/H <0.3. In the diffuse ISM mantle, heating by UV photons would graphitize the carbon mantles (${\mathrm{sp}}^{3}\to {\mathrm{sp}}^{2}$), which would then show a 3.3 μm emission feature [75]. All this is, in essence, incorporated and updated within the THEMIS model with the difference that the effects of dehydrogenation are assumed to be mediated by the effects of UV photolysis rather than by thermally driven, evaporative H atom loss.

## 3. Nanoparticles

A recent re-assessment of the composition and nature of interstellar carbonaceous nanoparticles resulted from a calculation of their chemical, structural and optical properties, i.e. a derivation of the size and surface dependence of their complex refractive indices [4,76,77] and the use of these within a new evolutionary interstellar dust model [13]. Perhaps, one of the major conclusions from these works is that the interstellar nanoparticle observables, i.e. far-UV (FUV) extinction, the UV bump, the IR emission bands and the MIR emission continuum, that had previously been attributed to different populations, are actually the various manifestations of the size-dependent properties of a single population of hydrogenated amorphous carbon, a-C(:H), nanoparticles (radii 0.4 to ∼20 nm). Here, the term a-C(:H) is used to encompass the entire family of aliphatic-rich, H-rich (a-C:H) to aromatic-rich, H-poor (a-C) hydrogenated amorphous carbon semi-conducting materials [78–80]. In fact, the above-mentioned evolutionary dust model [13] requires a large fraction (≈60%) of the carbonaceous material dust mass to be in particles with radii less than 20 nm. Thus, as it has long been recognized [6,7], hydro-carbonaceous nanoparticles would appear to be a particularly important constituent of interstellar dust and one that evolves in response to its local environment.

The nature of a-C(:H) nanoparticles and their evolution in circumstellar regions was explored within the context of fullerene formation in planetary nebulae [81,82]. The structure of these particles at nanometre size scales is rather interesting because they are an intimate and amorphous mix of aliphatic, olefinic and aromatic carbon structures [4,82]. The smallest of them, the so-called ‘arophatic’ particles [82], contain perhaps only a hundred or so carbon atoms and consist of small aromatic domains containing only a few rings (*N*_R_=1–3) that are linked into a contiguous network by relatively short chains (≈4 C atoms in length) of bridging aliphatics and/or olefinics [4,76,77,83]. The consequences of the chemical, structural and physical properties of these structures are explored in the rest of this paper.

### 3.1 Asphaltenes as a guiding framework

Building upon the recently presented ideas on the origin of the diffuse interstellar bands [64], it appears that poly-heterocyclic structures within interstellar a-C(:H) nanoparticles could play a key role in their chemistry within the ISM. This work [64,65] profits, in no small part, from the detailed analyses of the asphaltene moieties (extracted from petroleum and coal), which were studied using atomic force microscopy, molecular orbital imaging and scanning tunnelling microscopy [84]. These asphaltene moieties were shown to exhibit a wide range of complex aromatic structures, with no two having the same structure, and often with methyl and large alkyl peripheral side groups. While sixfold aromatic rings are predominant in the analysed species, they also contain a significant fraction of fivefold rings and rare sevenfold rings. These experiments also revealed the presence of a significant number of hetero-atom groups associated with the fivefold rings, which could be of CO, N, NH or S. Typically, asphaltene structures consist of a single aromatic core with alkyl side groups, which may be up to ≃2 nm (≃15 C atoms) long [84]. In §5, we comment on the structural similarities between the aromatic-rich moieties in terrestrial asphaltenes and biologically important porphyrin-based molecules such as chlorophyll and, in turn, their similarities to the aromatic-rich moieties probably present within the abundant aromatic carbonaceous grains in the ISM.

### 3.2 The kangaroo’s tail and the jelly bowl scenario

The stability of nanoparticles, and more generally of nanostructures, against the destructive effects of FUV photon absorption in the tenuous ISM is a matter that has perhaps not previously been given sufficient consideration. For example, as discussed in §(a), carbonaceous nanoparticles will almost certainly be significantly dehydrogenated by FUV photon-driven photolysis in the diffuse interstellar medium [59] but they can also dissociate as a result of the excitation arising from energetic (FUV) photon absorption [58,60,61]. However, and as discussed in the accompanying and following paper [64], there are several classes of species with low-lying electronic excited states from which efficient cooling is possible through fluorescent emission [85–87]. This fluorescent emission process is observed for $C_{6}^{-}$ [86,88,89], C_6_H^−^ [89], and is also seen in graphene, C_60_ anions, PAH cations and polyenes, which would make such species more resistant to photo-dissociation in the ISM than expected. Nevertheless, and whatever the final outcome of the excitation, nanoparticles will be significantly vibrationally excited by the absorption of energetic UV photons but could use at least some of this excitation energy to re-configure their structure to a different and/or more stable state [82], a well-known effect in irradiated materials [90]. A re-structuration upon photon absorption occurs in polyene-based molecules such as the carotenoids *β*-carotene and lycopene, azobenzenes, retinal (the molecule at the heart of vision) and diarylethenes (e.g. 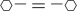). All of these polyene-containing molecules can, upon photon absorption, use the absorbed energy to flip their structure, between *cis* and *trans* forms, by rotation around a C=C *double* bond in their conjugated chains. Similarly, the formic acid molecule, $H-C\leq_{O}^{\mathrm{OH}}$, can undergo a *cis–trans* conformational switch, around the *single* C−OH bond, a process that has recently been observed in the ISM [91]. Thus, there do exist channels that can evacuate excess energy from a nanoparticle and therefore increase its photo-stability. In this sense, it may be that the aliphatic/olefinic bridges within the contiguous network of an a-C(:H) nanoparticle, and similarly the alkyl side groups on asphaltene moieties, can perhaps give an extra degree of stability to these mixed aromatic/olefinic/aliphatic nanospecies (as a kangaroo’s tail does to a kangaroo) by providing an absorber to dampen the destructive effects of large energy fluctuations. However, in the ISM it is likely that asphaltene-type alkyl tails are less stable than the bridging groups present within a-C(:H) nanoparticles [83] and that they would be removed and dehydrogenated during top-down photo-processing to perhaps form DIB-carrying polyene species [64]. In this sense, conjugated polyenic bridges (−HC=CH−HC=CH−HC=CH−), derived from the dehydrogenation of mixed aliphatic-olefinic structures [83], could introduce enhanced stability. Their low-lying electronic excited states would allow for cooling via fluorescent emission and, additionally, some of the absorbed energy would drive *cis* ↔ *trans* structural transformations. Increased internal energies (due to absorbed photons) will then probably flip the floppy aliphatic/olefinic bridges into new structural states, a process that is not possible with the more rigid aromatic domains. The known *cis–trans* photo-switching in polyene-containing species is driven by the absorption of visible photons (*E*=2–3 eV). However, an equivalent transformation in aromatic species would necessitate breaking aromatic bonds, requiring the localization of greater than 5.4 eV in a single $\text{C}\dddot{-}\text{C}$ bond (table 1), which is unlikely because of rapid internal vibrational re-distribution (10^−12^–10^−10^ s) [92]. Thus, the energy of absorbed FUV photons can probably be channelled into the internal transformation of aliphatic bridges into conjugated olefinics [83] leading to nanoparticle sub-structures with low-lying excited states that would then allow energy-shedding via fluorescent emission. However, at very high internal energies the particle will have few energy loss options other than dissociation, i.e. by fragmenting [60,83]. This is a bit like a glass bowl full of jelly; below some threshold tapping or shaking the bowl only makes the jelly wobble but shaking it above threshold deposits too much energy into the system and the glass bowl fragments spraying jelly blobs all over the place (e.g. when the bowl of jelly is dropped!).

**Table 1.** Some typical X−H and C−X bond energies, where C−H represents an aliphatic CH bond, =C<^H^ an olefinic CH bond and C−^⋯^ C an aromatic CC bond in benzene.

bond	bond energy (eV)	bond	bond energy (eV)
C−H	4.5	C−S	2.8
=C<^H^	4.8	C−N	3.2
N−H	4.0	C−C	3.6
O −H	4.8	C−O	3.7
		C−^⋯^ C	5.4
		C=S	5.9
		C=C	6.4
		C=N	6.4
		C=O	8.3
		C≡C	8.6
		C≡N	9.2

## 4. Nascence

Nascent nanoparticles generally exhibit enhanced surface reactivity due to large strain gradients which effect their geometry and induce confinement at the nanometre-scale [93]. For example, recent work shows that iron nanoparticles rust orders of magnitude faster than macroscopic particles [93] and gas phase CO_2_, SO_2_ and NO_2_ molecules react readily and reversibly with, respectively, 5, 50 and 100% of surface OH groups^2^ on titanium dioxide nanoparticles at 298 K [94]. These are but two examples of the vast multitude of work that has been undertaken in exploring the enhanced chemical reactivity at nanoparticle surfaces.

The enhanced surface activity observed in recently formed or nascent nanoparticles is of particular interest. However, this over-activity often tends to decay with time as the particle surfaces relax but it can also be reversible [94]. In the ISM where dust particles may exist for perhaps hundreds of millions of years, it would hardly seem that such particles could be considered as nascent. However, what is critical in determining whether nanoparticles are nascent is not their lifetime but the characteristic time-scale for their interaction with the local environment, i.e. with the gas of the surrounding medium in the case of the ISM. For example, in the tenuous or diffuse ISM (*n*_H_∼10^2^ cm^−3^) the typical interaction time-scale between an H atom and a nanoparticle is of the order of a year but for heavy atoms is typically 10^4^ yr (see the following section). Thus, in the low-density ISM a nanoparticle could perhaps react with typically only a few thousand heavy atoms (or a few million H atoms) during its lifetime there (∼10^7^ yr), which would appear to be comparable to the exposures that nascent nanoparticles are subjected to in laboratory experiments where the effects of active surface chemistry have been explored. However, in the low-density ISM the particles are bombarded with destructive photo-dissociating FUV photons, which will tend to cleanse the particles but will also destroy them. Thus, the interstellar nanoparticle population could be considered as nascent because it is most likely in a state of top-down flux with new particles being continuously created from larger grains [20,13,64,95,96]. In less destructive, denser interstellar media (*n*_H_≫10^4^ cm^−3^), this no longer holds true because, while nanoparticles are more stable in these regions, their surfaces will become saturated and inactivated due to mantle accretion and by their coagulation onto larger grains.

In the low-density ISM, nanoparticles carry a substantial fraction of the total grain surface area^3^ available for surface chemistry and are therefore expected to play a critical role in interstellar chemistry. In most interstellar chemistry studies, the grain surfaces are treated as passive substrates providing sites for reactions between adsorbed species or between adsorbed and incident gas phase species, which may be atoms, ions, radicals or molecules. This is the classical scenario for molecular hydrogen formation on grain surfaces in the ISM [1,2]. However, a-C(:H) nanoparticle surfaces are chemically (re)active [83] and therefore able to interact with incident species in ways that have not yet been considered in interstellar chemistry studies, particularly in the diffuse/translucent ISM interfaces at the onset of molecular cloud formation. Thus, nascent interstellar nanoparticles could be an important driver of interstellar chemistry in transition regions where they are abundant, the gas density is high enough for a significant flux of gas phase species onto their surfaces and the interstellar UV radiation field can also play a role. For instance, nanoparticle surfaces will preferentially react with abundant gas phase species such as H, O and N atoms and Mg^+^, Si^+^, Fe^+^ and S^+^ ions in diffuse/translucent regions. Given that H atoms are the most abundant lightweights in the diffuse atomic ISM, we consider them separately and then consider the surface chemistry with the heavyweights.

### 4.1 H atom interactions and far-UV photolysis

The evolution of carbonaceous dust in the more tenuous regions of the ISM is probably driven by H atom interactions leading to CH bond formation, which is counterbalanced by CH bond dissociation by FUV photolysis [4,22,24,25,83,97–99]. In the diffuse ISM, the carbonaceous nanoparticles must be aromatic-rich (H-poor) in order to explain the 3–13 μm IR emission bands and the dust thermal emission at MIR-mm wavelengths [4,13,77,100]. If we make the assumption that interstellar carbonaceous nanoparticles are optically thin to photon absorption at FUV wavelengths [4] and are collisionally thin to interactions with H atoms incident from the gas, i.e. the H atoms can interact with any available adsorption sites that might be available to form CH bands without hindrance. Then, the key parameter is the ratio of the CH bond FUV photo-dissociation rate to the CH bond formation rate by H atom addition. The latter rate is given by

4.1 $$R_{H}≃n_{H}\sigma_{f}v_{H},$$

where *n*_H_ is the gas phase H atom density in the tenuous ISM (typically ≃30 H atoms cm^−3^), *σ*_*f*_ is the CH bond formation cross-section (≃2×10^−18^ cm^2^) [25] and *v*_H_ is the H atom speed (*v*_H_=[8*k*_B_*T*_kin_/{*πm*_H_}]^0.5^=1.3×10^5^ cm s^−1^ at *T*_kin_=80 *K*≡0.01 eV). The counteracting CH bond FUV photo-dissociation rate is

4.2 $$R_{\mathrm{diss}.}≃F_{\lambda }\;e^{-\tau_{\lambda }}\sigma_{\mathrm{CH}},$$

where *F*_λ_ is the FUV photon flux (3×10^7^ photons cm^−2^ s^−1^) [101], *e*^−*τ*_λ_^ is the attenuation of the FUV radiation field at optical depth *τ*_λ_ (hereafter assumed to be zero for the tenuous and optically thin diffuse ISM) and *σ*_CH_ is the CH bond FUV photo-dissociation cross-section (≃10^−19^ cm^−2^) [22,97,98,102,103]. The critical ratio of these two rates, *ζ*, is then

4.3 $$\zeta =\frac{R_{\mathrm{diss}.}}{R_{H}}=\frac{F_{\lambda }\sigma_{\mathrm{CH}}}{n_{H}\sigma_{f}v_{H}}.$$

If we substitute the above-cited values into equation (4.3), we find *ζ*≃0.4, seemingly slightly favouring hydrogenation over photolysis. However, it should be noted that while *σ*_CH_ has been measured for methane molecules [103], which is probably a good approximation for the CH bonds in a-C(:H) nanoparticles, the value of *σ*_*f*_, the CH bond formation cross-section, was obtained for bulk materials rather than nanoparticles. It is difficult to estimate the uncertainty in the above rates because of the few relevant cross-section data available for comparison and the uncertainties in the interstellar FUV photon flux. With this limitation in mind, if the rates *R*_H_ and *R*_diss._ are similar (i.e. *ζ* of order unity) H atoms would be lost at about the same rate as they are (re-)incorporated and the nanoparticles would be about 50% dehydrogenated at equilibrium. Further, while all a-C(:H) nanoparticles probably contain photolysable CH bonds they may not always express suitable sites for H atom addition, i.e. the efficiency for (re-)hydrogenation may be intrinsically lower than that for CH bond photolysis. Given that the observational evidence points to the dominance of aromatic-rich (nano)particles to explain the dust IR-mm emission features and continuum in the tenuous ISM, where there is little extinction, it would seem that FUV photolysis is favoured over the effects of hydrogenation [4,13,26] but that with increasing extinction the balance would shift in favour of hydrogenation [100].

### 4.2 Interaction with the heavies

This sub-section focuses on the formation of diatomic hydrides, in particular the hydroxyl radical, OH, but also the imidogen radical, NH (also called, hydridonitrogen, aminylene or nitrene), in the tenuous, low-density, atomic ISM. Since its discovery more than 50 years ago [104], OH has been of particular interest to astronomers because, after H_2_, it is the second most abundant molecule in space and so, for many, the discovery of OH in 1963 really marks the birth of interstellar chemistry. Nevertheless, it was not until 2010, almost 50 years later, that the cation, OH^+^, was first detected in the interstellar medium [105].

The currently accepted route to OH formation in the ISM is initiated by the reaction of cosmic ray-formed H^+^ or H${\;}_{2}^{+}$ with atomic oxygen to form OH^+^. The route to OH is then thought to proceed through the reaction sequence [106,107]: OH^+^ (H_2_:H) H_2_O^+^ (H_2_:H) H_3_O^+^ (e^−^:2H) OH *or* (e^−^:H) H_2_O *or* (e^−^:H_2_+H) O where we adopt the descriptor *primary reactant (reactant:product) primary product*. Of the three possible branchings in the dissociative recombination of H_3_O^+^, the last step in the OH formation chain, more than 80% of the possible outcomes favour OH formation [108,109]. Recent observational work on interstellar oxygen-containing species in diffuse clouds [107] underlines the notion that the first-step reaction, i.e. OH^+^ (H_2_:H) H_2_O^+^, represents a bottleneck in the formation of interstellar OH. We note that abundant OH is observed in diffuse, atomic cirrus clouds [110,111], regions almost devoid of molecular hydrogen, it would then appear that there is perhaps a cork in this bottleneck reaction and therefore that the formation of OH in atomic clouds is somewhat problematic.

Given that heavy atoms (*O*,C,*N*,…) are about four orders of magnitude less abundant than H atoms in the gas in the diffuse ISM, it is clear that for them *ζ*≫1 (see equation (4.3)) and so, during their surface residence time, an adsorbed/chemisorbed heavy atom X will be subject to a significant number of UV photon absorption events that will affect its surface residence time and adsorbed state, and could drive the catalytic (dissociative) formation of small radicals. The intrinsic structure of an a-C(:H) nanoparticle includes domains with aliphatic, olefinic and aromatic C−H and C−C, C=C bonding, of which the unsaturated olefinic, and to a lesser extent the aromatic, C=C bonds will be receptive to adsorptive chemical bond formation with incident gas phase atoms (O, C, N, …). Table 1 gives the typical energies of some of the bonds likely to be formed between the most abundant interstellar atoms.

**Figure 1.**
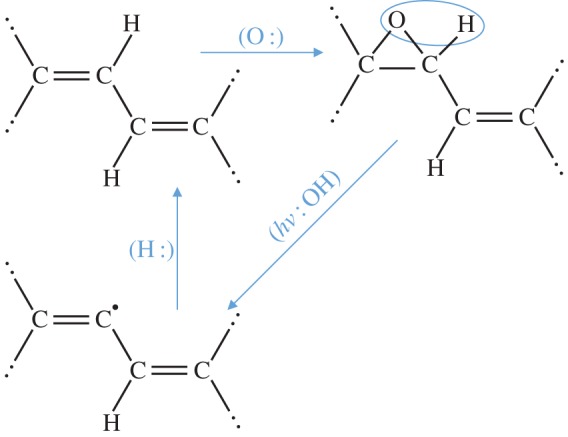
A formation pathway for nanoparticle surface epoxide groups and the catalytic formation and liberation of OH radicals following UV photolysis in the tenuous ISM. Note that in this and the following three figures the species denoted with double-dotted bonds are assumed to be bonded to the contiguous structure of the nanoparticle.

**Figure 2.**
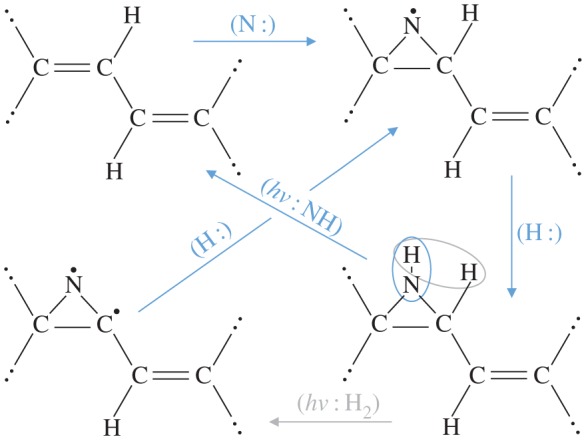
A formation pathway for nanoparticle surface aziridine groups and the catalytic formation and the liberation of H_2_ molecules and NH radicals following UV photolysis in the tenuous ISM.

In the diffuse ISM reactions with the neutral, O and N atoms and C^+^ ions will be favoured because of their high gas phase abundances. Further, and given that the smallest and most abundant nanoparticles will be predominantly neutral [112], reactions with the less abundant ions Mg^+^, Si^+^, Fe^+^ and S^+^ will not be hindered. For example, it is highly probable that O and N atoms will react with the olefinic double bonds, >C=C<, that link the aromatic domains, rather than with the more stable aromatic C=C bonds, in a-C(:H) nanoparticles [83]. These reactions will form epoxide-type groups with O atoms [113] and aziridine-type groups with N atoms, the latter being the nitrogen analogue of the epoxide group. Using the bond energies (table 1), the formation of an epoxide (aziridine) group by this route would appear to be favoured by ≈5 eV (≈4 eV). In each of these chemical species, the fundamental structure consists of a C_2_X three-membered ring >C^X^_−_C< (X=O or N), which is very reactive and readily decomposes upon exposure to UV. Thus, the epoxide and aziridine groups could be key intermediates in the photon-driven formation of small radicals in tenuous interstellar clouds. In such regions, the most likely products of the stellar UV photolysis of epoxide- and aziridine-type groups would be OH and NH via the following reaction pathways, where the X indicates either O or N atoms:

$$\begin{array}{rl} & {\;}^{H}>C\text{=}C<\;\to (↓X:)\to^{H}>C_{-}^{X}C<\;\to (h\nu^{⋆}:{\;}^{↑}\mathrm{XH})\to \;>C\text{=}C<\;\to (H:)\to^{H}>C\text{=}C<\\ & \;>C\text{=}C<\;\to (↓X:)\to \;>C_{-}^{X}C<\;\to (↓H:)\to \;>C_{-}^{X^{H}}C<\;\to (h\nu^{⋆}{:}^{↑}\mathrm{XH})\to \;>C\text{=}C< \end{array}$$

with *hν*^⋆^ being reaction-driving UV photons. The likely pathways for the formation of nanoparticle surface epoxide and aziridine groups and their subsequent photolysis/surface catalysis routes to OH and NH radical formation, and molecular hydrogen formation via an aziridine, are shown in more detail in figures 1 and 2. Using the bond energies in table 1, it would appear that the decompsition of epoxide (aziridine) groups to form OH (NH) is disfavoured by ≈8 eV (≈7 eV), including the release of the ≃1 eV of strain energy tied up in the ‘spring-loaded’ three-membered ring.^4^ However, as is well known, epoxides readily decompose upon exposure to UV photons and so their decomposition in the diffuse ISM, where is there is no lack of 7–10 eV UV photons, is unlikely to be a barrier to OH and NH formation.

A point of concern with this proposed mechanism is that FUV photolysis might not necessarily release OH (NH) radicals from the epoxide (aziridine) rings. Given that epoxide ring-opening reactions, involving acidic (H_3_O^+^ or H^+^) or basic (OH^−^) reagents, always lead to hydroxyl bond formation in alcohols and diols, the formation of OH groups on/within a-C(:H) nanoparticles would seem to be assured. The question is then, what is the fate of epoxide rings and any subsequently formed hydroxyl groups upon photolysis? It has been shown experimentally that UV light causes both of the epoxide ring C−O bonds to break and that the resulting intermediate radicals (an O atom and the >C^•^−^•^C<^H^ group) then re-arrange to form alcohols, aldehydes or ketones [114]. Thus, it would appear that an intermediate radical O atom, produced as a result of UV photolysis of an epoxide ring, will seek out a suitable neighbouring reaction partner, which in a-C(:H) nanoparticles is most likely to be an H atom.^5^ The so-formed OH radical then has a non-negligible probability of being ejected from the nanoparticle before it can find a suitable binding site on the grain.

In an analogue to the FUV photolysis of CH bonds in a-C(:H) nanoparticles [83], the OH and NH bonds in epoxide and aziridine groups, on the surfaces of and within such particles, will also be subject to FUV photolysis in the ISM. This should occur at a rate similar to that for gas phase OH photo-dissociation, i.e. *R*_diss.(OH)_≃2×10^−10^ s^−1^ [115]. However, as per CH bond photolysis, this rate will be reduced by the nanoparticle absorption efficiency factor at FUV wavelengths, *Q*_abs_(*a*,λ_FUV_), i.e. not all of the incident FUV photons are absorbed by the nanoparticle, and also by an additional factor (*ϵ*) because, depending on grain size, not all photons absorbed by the particle will necessarily lead to OH bond photolysis [100,83]. Following our previous work [4,83,100], *Q*_abs_(*a*,λ_FUV_)≤0.1 [4] and *ϵ*≃1 for nanoparticles with radii ≤1 nm [100,83]. Thus, the epoxide OH bond photolysis rate, and by analogy that for aziridine NH bonds, within nanoparticles is *R*_diss.(OH)_*Q*_abs_(*a*,λ_FUV_)*ϵ*≃10^−11^ s^−1^, i.e. about an order of magnitude lower than that for gas phase OH and NH radicals.

The rate-determining step in the formation of OH and NH via the above epoxide and aziridine pathways, respectively, is the X atom (O or N) chemisorption rate onto nanoparticles, which is given by

4.4 $$R_{X}=n_{H}\;X_{X}\;\pi a_{\mathrm{np}}^{2}\;v_{X}\;s_{X},$$

where *n*_H_ is the H atom density, X_X_ is the gas phase abundance of atom X relative to hydrogen, *v*_X_ its thermal speed and *s*_X_ its sticking efficiency. Adopting, *a*=0.4 nm for a-C(:H) nanoparticles in the atomic ISM [13], *n*_H_=30 cm^−3^, X_O_=465 ppm (cosmic abundance of 575 ppm [116] with 110 ppm depleted into silicate dust [71]), X_N_≃64–100 ppm [117,118], *s*_O_=*s*_N_=1,^6^ and *v*_O_=*v*_N_≃3×10^4^ cm s^−1^, the formation rate for OH is then *R*_OH_=2.1×10^−12^ s^−1^. For NH the formation rate will probably be at least a factor of five less than that for OH, given the nitrogen relative abundance and all other parameters equal. However, nitrogen appears to have an affinity for a-C(:H) materials and appears to promote sp^2^ cluster formation [119–122]. Hence, once incorporated into a-C(:H) nitrogen atoms may be harder to remove by FUV photolysis than epoxide-bonded oxygen atoms, and so NH formation by this route is likely to be intrinsically less efficient than OH formation. The derived OH and NH formation rates will probably hold for a-C(:H) nanoparticles (a≲1 nm) in the diffuse/translucent ISM with low extinction, i.e. $A_{V}≲0.5\;\mathrm{mag}.$, where the UV photon flux is greater than the O and N atom fluences.

In the ISM surface-epoxide-formed OH will be photo-dissociated, at a rate *R*_diss._≃2×10^−10^ s^−1^ [115], once released into the gas phase in low extinction regions ($A_{V}≲1\;\mathrm{mag}.$). From the reaction kinetics, we have that

4.5 $$\frac{dn\left[\mathrm{OH}\right]}{dt}=-R_{\mathrm{diss}.}\;n_{\mathrm{OH}}+R_{\mathrm{OH}}n_{H}X_{\mathrm{np}},$$

where X_np_ is the nanoparticle abundance relative to hydrogen. For the almost mono-modal a-C(:H) nanoparticle size distribution in the low-density atomic ISM X_np_≃5×10^−6^ for *a*=0.4 nm (≃150 ppm of carbon out of a total dust budget carbon requirement of ≃200 ppm) [13]. The above-derived OH formation rate, $R_{\mathrm{OH}}=2.1\times 10^{-12}\;s^{-1}$, along with X_O_=465×10^−6^, then implies an OH relative abundance in these regions given by *n*_OH_/*n*_H_=(*R*_OH_ X_np_)/*R*_diss._∼5×10^−8^. For the formation of NH, using the same photo-dissociation rate as for oxygen, but assuming that some fraction, *f*_N,*np*_, of the nitrogen atoms become trapped into aromatic moiety peripheral sites in nanoparticles, we find $n_{\mathrm{NH}}/n_{H}≲(1-f_{N,\mathrm{np}})\times 10^{-9}$ and suggest that the NH abundance will be at least an order of magnitude less than that of OH in a given region.

In the tenuous or translucent ISM, OH appears to be rather widespread and to follow atomic hydrogen (HI), both spatially and in velocity extent, rather than CO [110,111]. OH appears to correlate with the dust long-wavelength emission at 100 μm and to increase in abundance with the visual extinction above a threshold of $A_{V}≳0.5\;\mathrm{mag}.$ ($\equiv N_{H}≳(2.2\text{--}2.9)\times 10^{20}\;{\mathrm{cm}}^{-2}$; similar to the observed threshold for H_2_ formation, i.e. $N_{H}≳(2.9\text{--}3.6)\times 10^{20}\;{\mathrm{cm}}^{-2}$) [110]. The abundance of OH relative to atomic HI observed in the cirrus clouds in the North Celestial Loop is *N*_OH_/*N*_HI_=(6.5–7.4)×10^−8^ [110]. The above-estimated photolysis-driven OH relative abundance (5×10^−8^)^7^ for the low-density ISM therefore appears to be in reasonable agreement with the observed OH abundances. The fundamental process underlying this proposed photolytic OH formation route is compatible with a similar mechanism for the photolysis-driven formation of H_2_ in moderately excited photo-dissociation regions [83].

**Figure 3.**
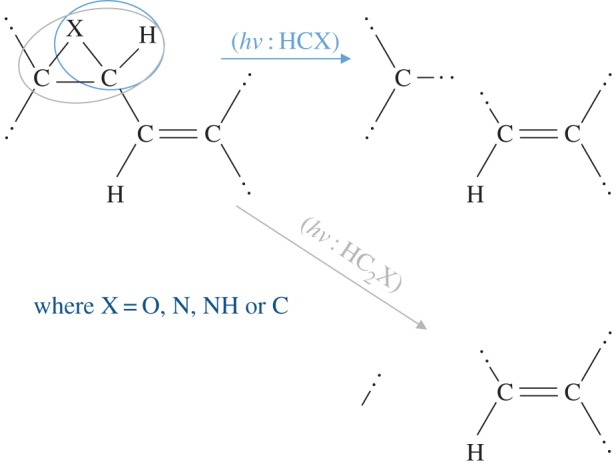
A speculative epoxide/aziridine FUV photolysis-driven pathway to nanoparticle erosion in the tenuous ISM. In this case, the product radicals could include: HCO, HC_2_O, HCN, HNC and HCNH and perhaps also HC_2_NH, l-C_3_H and c-C_3_H if C^+^ inserts into olefinic C=C bonds to form a cyclopropene ring, somewhat analogous to epoxide and aziridine formation.

In the above, it was assumed that the heavy atom incorporation into nanoparticles and the resultant catalytic formation of OH and NH radicals is recyclable, i.e. there is no inherent destruction of the grain structure. However, and given that things seem to be rather finely balanced in the tenuous ISM, it is possible that the scales could be tipped towards FUV photolysis leading to the loss of radical species with two heavy atoms, therefore resulting in nanoparticle erosion. A postulated a-C(:H) erosional pathway is illustrated in figure 3, which indicates that some interesting radical species could be liberated into the gas where they would almost certainly be ionized and dehydrogenated. From bond energy considerations (table 1), and including the ≃1 eV of strain energy in the epoxide ring, these nanoparticle-destroying reactions would appear to be disfavoured by some 5–7 eV, which is well within the typical FUV photon energies (*E*_hν_≃6–12 eV) available within tenuous and diffuse interstellar media. Thus, such erosional photolytic reactions can therefore only occur in low-density, low-extinction regions ($A_{V}≲0.5$, $n_{H}≲10^{2}\;{\mathrm{cm}}^{-3}$) of the ISM.

## 5. Nitrogen

Given the affinity of nitrogen for incorporation into a-C(:H) materials [119–122], a possible key role for nitrogen hetero-atoms within interstellar carbonaceous grains has been considered within the astrophysical context [19,64,123] and from this work it appears that a doping level only of the order of 1% or so, with respect to carbon, would yield interesting effects [19,123].

Nitrogen atoms can be incorporated into a-C:H materials (designated a-C:N:H) but only rather inefficiently [124] and any observable effects of its presence are removed by heating to ≈800 *K* [125]. Within the a-C:H structure N atoms, incorporated in C−N and C=N bonds, tend to promote the formation of sp^2^ carbon clusters, i.e. aromatic domains, within the network [119–121] and decrease the optical gap [124,120]. It is noteworthy that a-C:H materials with a nitrogen atom fraction as low as 1% and an optical gap of ≃1 eV are rich with reactive dangling bonds and exhibit conductivity in the valence band tail. However, as the N atom fraction increases a-C:N:H becomes more polymeric, forming >C=N−, and the structure connectivity decreases as the concentration of network-terminating −C≡N and −NH_2_ groups increases [126]. Interestingly, nitrogen doping also leads to symmetry-breaking and the activation of olefinic >C=C< stretching modes in a-C:N:H [119]. Further, its incorporation into aromatic domains leads to ferro-magnetism in a-C:N:H [127], which could perhaps have interesting implications for the origin of the anomalous microwave emission, attributed spinning nanoparticles, and the polarization of interstellar carbonaceous grains.

Despite an apparent lack of observational evidence for nitrogen in interstellar hydrocarbon dust [128,129], it could still be present because the CC and CN infrared band positions in aromatic clusters are similar [80], making the detection of any nitrogen atom doping of interstellar carbonaceous dust difficult. However, the nitrogen doping of a-C:H only leads to rather subtle spectral changes, which appear as broad bands at 6.17 and 8 μm, and as a broadening and variation in the relative intensities of the CH_2_ and CH_3_ bands [125]. Thus, the detection of nitrogen in interstellar carbonaceous dust, through spectroscopy in the 3–10 μm wavelength range, will not be easy [125]. Nevertheless, and given that the doping of interstellar a-C(:H) nanoparticles with nitrogen could be an important element in our search for an explanation for the nature of the DIB carriers [19,64,123], any efforts in this direction might eventually prove worthwhile.

**Figure 4.**
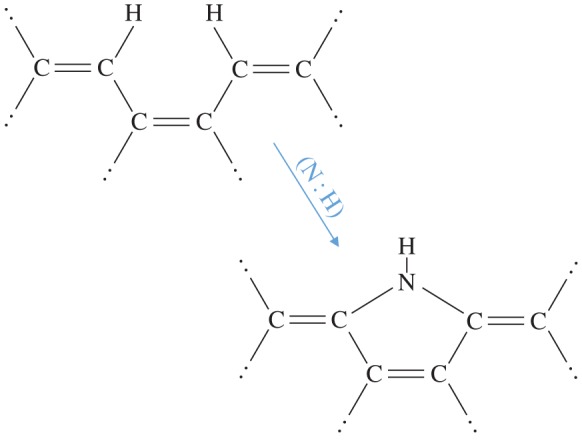
A route to N-containing, hetero-atom, fivefold rings in poly-olefinics (polyethenes) and on the periphery of aromatic moieties.

**Figure 5.**
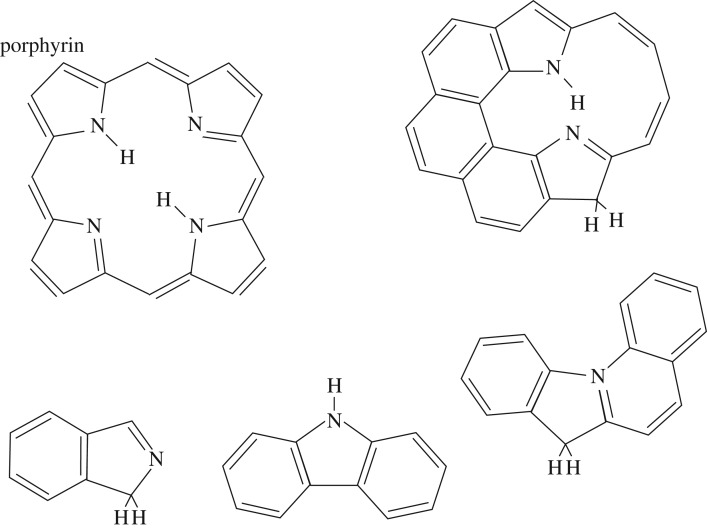
The porphyrin chlorin ring structure (upper left) and possible porphyrin precursor moieties typical of asphaltene-type structures and aromatic domains within N-doped a-C(:H) nanoparticles (increasing complexity from bottom left to upper right).

Given that nitrogen has a propensity for incorporation into aromatic domains, it is likely that N-doped a-C(:H) cage structures, fullerene-type structures and cage-trapped metals could provide a route to N-containing hydrocarbon clusters analogous to porphyrin-type species, where the nitrogen atoms become preferentially trapped in fivefold rings as appears to be a common feature of asphaltene-type aromatic moieties [84,64]. Indeed, a possible route to peripheral, nitrogen-containing, fivefold rings on olefinics and aromatics is shown in figure 4, which would appear to be favourable by about 0.8 eV, given the typical bond energies involved (table 1). Further, these types of structures, as schematically shown in figure 5, could provide a stepping stone to the formation of the precursors of the chlorin structures in porphyrin-type moieties. Porphyrins are key elements in the structure of chlorophyll and heme when associated with centrally coordinated magnesium and iron atoms, respectively. Interestingly, porphyrin structures exhibit a strong blue absorption band in the approximately 430–450 nm wavelength region, which is intriguingly close to the strongest DIB at 443 nm. Indeed, porphyrins would appear to have many bands in common with the DIBs [130].

Chlorophyll molecules (C_35_H_28–30_O_5_N_4_Mg, type c1 and c2; C_54–55_H_70–72_O_5–6_N_4_Mg, type a, b, d and e) are basically aromatic-type clusters with a magnesium atom at their centre and a long alkane chain about 15 C atoms long, with ether bridges and carbonyl groups at its base. Likewise within haemoglobin the heme structure (C_34_H_32–36_O_4_N_4_S_0–2_, type b or c; C_49_H_55–56_O_4–6_N_4_Fe, type a or o) has a porphyrin structure with a central iron atom and carboxylic acid-terminated alkyl side groups. In chlorophyll, the extended alkyl chain’s primary role appears to allow the molecule to incorporate within hydrophobic environments. However, molecular dynamics simulations show that in water the molecule adopts a more compact structure with the chain folded onto the chlorin ring (upper left structure in figure 5) [131]. Thus, the alkyl chain is not necessarily a pendant structure but can wrap around making the chlorophyll molecule into a spheroidal nanoparticle containing approximately 70 atoms. Apart from this, other possible roles for the alkyl chain do not seem to have been given much consideration. The long alkane chain, with carbonyl and ether groups at its base, is indeed a rather curious beast and it appears that such a structure would provide an ideal energy absorber to enable the molecule to dissipate the energy associated with photon absorption through vibrational excitation. Hence, the alkyl chain may have evolved into an energy absorber that stabilizes the molecule against excitation in the same way that the aliphatic/olefinic bridges within the contiguous networks of a-C(:H) may play an energy-absorbing role in interstellar nanoparticles (see §§(a)and (b)). It is interesting that long alkyl side groups, similar in length to those of chlorophyll molecules, are a rather common feature of the analysed asphaltenes extracted from coal and oil [84]. Thus, the chlorophyll structure appears to bear a generically rather striking resemblance to the structures observed within asphaltene moieties. This is perhaps not too surprising given that asphaltenes originate from the decay of organic plant matter, a process that has occurred over geological time-scales.

## 6. Natural selection

Interstellar a-C(:H) nanoparticles with up to a few hundred heavy atoms can probably exist in tens of millions of different configurational forms [64]. It is interesting that the key porphyrin-based structures within the chlorophyll and heme molecules contain only of the order of ∼40–70 and ∼40–60 heavy atoms, respectively, which is considerably fewer atoms than a typical interstellar nanoparticle. Further, and as mentioned above, such generic types of structures are a feature of the naturally occurring asphaltene moieties.

Thus, extra-solar system nanoparticles incorporated into larger grains and small solar system bodies in the pre-solar nebula, raining down through a planetary atmosphere onto the surface, would provide a very rich resource from which to inorganically select the more stable and later-to-be-useful species. A selection process would likely occur through photo, thermal and aqueous processes operating over geological time-scales. Thus, viable solar energy absorbing structures (aka, chlorophyll) and trappers and transporters of useful molecules within and across particle boundaries (aka, O_2_, CO transport by haemoglobin) could have been selected over long time-scales to provide a source of pre-precursor, pre-biotic molecules that would act as complex chemistry drivers on the primitive Earth. These reactions would eventually lead this chemistry along a route to the first simple self-replicating biotic species. Thus, while life may not have come from space it was probably seeded with a wide variety of species that would inevitably lead to the appearance of life on the Earth.

Thus, even before natural selection could operate on living organisms, the stage for it’s operation at the molecular level may already have been set long before any self-replicating structures or species appeared on Earth.

### 6.1 Implications

A route towards the precursor^8^ seeding of later-formed key (pre-)biotic molecules appears as though it could have been influenced by the intrinsic nature of pre-existing interstellar carbonaceous grains polluted with nitrogen, oxygen and other less-abundant hetero-atoms. Once these interstellar grains are transported to a planetary surface they at last find themselves in a protective environment shielded from the effects of harmful radiation in the ISM. On a planetary surface, these grains will provide a highly diverse and valuable feedstock from which the local conditions would down-select the most favourably stable structures for that given environment. This would most probably occur in the near-surface sediments of an aqueous environment, where the effects of the proto-stellar radiation field are suitably attenuated but sufficiently intense to provide an energy source for chemical reactions. Among these reactions simple ion-trapping by solids from the overlying solution (primeval soup?) could lead to the incorporation of metal cations within ‘selected’ precursor inorganics (aromatic-type moieties) of extra-planetary origin.

It then appears that the precursor species could provide a natural but slow route to pre-biotic species, including pre-porphyrins, that could through inorganic selection over the millennia have yielded some interesting chlorophyll-like and heme-like species. Thus, many inorganic molecules and structures, both simple and more complex, could have influenced the chemical pathways that would eventually lead to living organisms. Thus, natural selection is probably also an important mechanism, that operates at the molecular level in a primitive planetary surface environment, to weed out the less-than-useful chemical species and make the best use of stable species with ‘valuable’ properties. A major implication from this line of reasoning is that, given the same source materials of interstellar origin, the chemical and biological evolution leading to life probably follows similar pathways in similar environments.

## 7. Conclusion

The chemistry and physics of (interstellar) nanoparticles is very particular, in that important size-dependent effects have to be taken into account when modelling the effects of the environment on nanoparticles (evolution through erosion, mantling, coagulation, …) and their return effects on that environment (photo-electric heating of the gas, formation of molecular hydrogen and small radicals by surface reactions, …). Thus, a full understanding of their properties will be essential in advancing our understanding of the evolution of the low-density ISM.

In no small part, interstellar chemistry in transition regions, the clouds in the stages of evolution between the most diffuse clouds and molecular clouds, is probably driven by the active surface chemistry of carbonaceous, a-C(:H), nanoparticles. The catalytic FUV photon-driven formation of OH, NH and other small radicals and molecules (e.g. HCO, HC_2_O, HCN, HNC, HCNH, HC_2_NH, l-C_3_H and c-C_3_H, …) on and within nanoparticles may therefore be the initiator of the chemistry in the most tenuous regions of the ISM. Further, it is also likely that interstellar nanoparticles will retain their inherent reactivity whether in a free-flying state or coagulated into aggregates, provided that they are not submerged under an ‘inert’ molecular ice mantle.

Having a preference for incorporation within the fivefold hetero-cycles associated with aromatic-rich moieties, nitrogen is a key ingredient in many interesting molecules (e.g. porphyrins, chlorophyll, heme, …) where it is present at the level of only a few atomic per cent. The importance of nitrogen in dust, and particularly in nanoparticles, has therefore most likely been overlooked because of the low levels of depletion required to yield interesting effects. Small nitrogen depletions are hard to detect and, in any event, the direct association of nitrogen with any observable measure of dust in the low-density ISM has yet to be made.

It is likely that planetary surfaces will be seeded with precursor species from a universal source (interstellar and interplanetary medium dust, nanoparticles in particular). These particles will provide a rich chemical variety of nanometre-sized structures. Principal among them are likely to be analogues of the aromatic-rich moieties extracted from terrestrial coals and oils (asphaltenes). The principal effect of the planetary surface processing of this rich resource, most likely in shallow aqueous bodies, would be to naturally down-select the most stable species that could, over geological time-scales, yield useful pre-biotic molecules. If these seed species are a significant link in the chain of events leading to life, then the origin of life will most likely follow the same chemical pathways in similar environments. If favourable life-forming environments turn out to be rather restricted, i.e. to terrestrial-like planets, then perhaps life may be rather similarly constructed everywhere in the Universe, wherever and whenever it arises.

## Footnotes

1 Amorphous hydrocarbons are macroscopically structured, solid-state materials consisting of only carbon and hydrogen atoms: a-C:H materials are H-rich (15–60 at.% H), aliphatic-rich and wide band gap (more than or equal to 1 eV), whereas a-C materials are H-poor (less than 15 at.% H), aromatic-rich and narrow band gap (approx. −0.1 to 1 eV).

2 With CO_2_ this yields, among others, surface carboxylic acid, carbonate and bicarbonate species and to the sulfur and nitrogen analogues with SO_2_ and NO_2_, respectively [94].

3 For the THEMIS model, ∼98% of the total dust cross-section is to be found in the steep power-law distribution of a-C nanoparticles with radii 0.4–20 nm.

4 For example, see http://crab.rutgers.edu/∼alroche/Ch14.pdf.

5 In general in this type of reaction the epoxide O atom tends to ‘lean’ towards the least-substituted of the two carbon atoms in the epoxide ring (e.g. see http://chem.libretexts.org/Textbook_Maps/Organic_Chemistry_Textbook_Maps/Map%3A_Organic_Chemistry_With_a_Biological_Emphasis_(Soderberg)/Chapter_08%3A_Nucleophilic_substitution_reactions_I/8.6%3A_Epoxides_as_electrophiles_in_nucleophilic_substitution_reactions). Within an a-C(:H) network, the most likely CH bonding configurations would appear to be ^H^>C^O^_−_C< and ^H^>C^O^_−_C<^H^,[76] which means that there is always an H atom adjacent to an epoxide ring and therefore available for OH bond formation.

6 For large interstellar grains, i.e. bulk materials at low temperatures, a heavy atom sticking coefficient of unity is generally assumed. Such a high value seems unlikely for nanoparticles, which are typically only tens of atoms across, and in this case trapping might perhaps be a better description of the interaction. However, there do not appear to be any suitable measurements or simulation constraints for atom sticking or trapping by carbonaceous nanoparticles. In the absence of evidence to the contrary, we assume a sticking coefficient of unity for heavy atom interactions with nanoparticles. However, if the sticking/trapping coefficients are determined to be less than unity for nanoparticles then the chemisorption rates calculated here will need to be scaled down accordingly.

7 N.B., If the sticking/trapping coefficients for atom interactions with nanoparticles are determined to be less than unity then this rate will need to be scaled down appropriately.

8 Precursor in this sense is taken to mean the chemical species that pre-date biotic molecules by a few major steps, these being: biotic molecules derived from pre-biotic molecules (inorganic species of relevance to biology but not biologically active), which may themselves be derived from extraneous matter (extra-planetary system solid bodies).

## Data accessibility

Details of and the input data for the THEMIS dust model can be found at: http://www.ias.u-psud.fr/THEMIS/ and http://www.ias.u-psud.fr/DUSTEM/.
